# Multivalent Chromosomal Expression of the Clostridium botulinum Serotype A Neurotoxin Heavy-Chain Antigen and the Bacillus anthracis Protective Antigen in Lactobacillus acidophilus

**DOI:** 10.1128/AEM.01533-16

**Published:** 2016-09-30

**Authors:** Sarah O'Flaherty, Todd R. Klaenhammer

**Affiliations:** Department of Food, Bioprocessing and Nutrition Sciences, North Carolina State University, Raleigh, North Carolina, USA; Pennsylvania State University

## Abstract

Clostridium botulinum and Bacillus anthracis produce potent toxins that cause severe disease in humans. New and improved vaccines are needed for both of these pathogens. For mucosal vaccine delivery using lactic acid bacteria, chromosomal expression of antigens is preferred over plasmid-based expression systems, as chromosomal expression circumvents plasmid instability and the need for antibiotic pressure. In this study, we constructed three strains of Lactobacillus acidophilus NCFM expressing from the chromosome (i) the nontoxic host receptor-binding domain of the heavy chain of Clostridium botulinum serotype A neurotoxin (BoNT/A-Hc), (ii) the anthrax protective antigen (PA), and (iii) both the BoNT/A-Hc and the PA. The BoNT/A-Hc vaccine cassette was engineered to contain the signal peptide from the S-layer protein A from L. acidophilus and a dendritic-cell-targeting peptide. A chromosomal region downstream of *lba0889* carrying a highly expressed enolase gene was selected for insertion of the vaccine cassettes. Western blot analysis confirmed the heterologous expression of the two antigens from plasmid and chromosome locations. Stability assays demonstrated loss of the vaccine cassettes from expression plasmids without antibiotic maintenance. RNA sequencing showed high expression of each antigen and that insertion of the vaccine cassettes had little to no effect on the transcription of other genes in the chromosome. This study demonstrated that chromosomal integrative recombinant strains are promising vaccine delivery vehicles when targeted into high-expression chromosomal regions. Levels of expression match high-copy-number plasmids and eliminate the requirement for antibiotic selective maintenance of recombinant plasmids.

**IMPORTANCE** Clostridium botulinum and Bacillus anthracis produce potent neurotoxins that pose a biochemical warfare concern; therefore, effective vaccines against these bacteria are required. Chromosomal expression of antigens is preferred over plasmid-based expression systems since expressing antigens from a chromosomal location confers an advantage to the vaccine strains by eliminating the antibiotic maintenance required for plasmids and negates issues with plasmid instability that would result in loss of the antigen. Lactic acid bacteria, including Lactobacillus acidophilus, have shown potential for mucosal vaccine delivery, as L. acidophilus is bile and acid tolerant, allowing transit through the gastrointestinal tract where cells interact with host epithelial and immune cells, including dendritic cells. In this study, we successfully expressed C. botulinum and B. anthracis antigens in the probiotic L. acidophilus strain NCFM. Both antigens were highly expressed individually or in tandem from the chromosome of L. acidophilus.

## INTRODUCTION

Lactic acid bacteria (LAB) are promising candidates for the delivery of antigens, antiallergens, and other products to the mucosa and are not highly immunogenic (1). Lactobacillus acidophilus NCFM, a probiotic LAB strain safely consumed for nearly 40 years, has been shown to be a prime candidate for delivery of antigens to the intestinal mucosa, as it is bile and acid tolerant, allowing survival through the gastrointestinal tract. Significantly, L. acidophilus NCFM has been shown in numerous studies to interact with the immune system (2) and in particular with dendritic cells (DCs) through the DC-specific intercellular adhesion molecule 3-grabbing nonintegrin (DC-SIGN) (3). L. acidophilus has demonstrated efficacy for the expression of the Bacillus anthracis protective antigen (PA) in conjunction with a DC-targeting peptide (4). This 12-amino-acid peptide has previously been shown to target DCs, resulting in enhanced immunogenicity (4, 5). L. acidophilus has also been used successfully for cell surface display of HIV-1 Gag protein and the Salmonella flagellin FliC (6, 7). However, these studies utilized plasmid-based expression systems. Expressing antigens from a chromosomal location rather than from a plasmid confers an advantage to the vaccine strains by removing the need for the antibiotic maintenance required for plasmids and negating the chance of plasmid instability that would result in loss of the antigen. Additionally, from a production and regulatory point of view, omitting the need for antibiotic pressure is favorable when performing human clinical trials. High-expression regions of the L. acidophilus chromosome have been identified for the expression of a reporter enzyme (Gus3A) under the control of native promoters (8, 9). To date, this particular integrative food-grade-based expression system has not been utilized for the expression of antigens or biotherapeutics in L. acidophilus or other lactic acid bacteria. Using strains from the Lactobacillus group has many advantages: many of these strains are generally recognized as safe (including L. acidophilus NCFM), they facilitate the often-preferred mucosal route of vaccine administration, are resilient to gastrointestinal acids, provide natural adjuvant properties, and interact with cells of the immune system. Cells can be easily and inexpensively produced in large amounts. Additionally, these cells are routinely freeze-dried, stably stored, and distributed as oral supplements or delivered in dairy matrices, hence increasing the diversity of administration methods for vaccination purposes.

Clostridium botulinum and B. anthracis are Gram-positive, spore-forming bacteria commonly found in soil. Most members of the Clostridium and Bacillus genera rarely cause disease in humans; however, both C. botulinum and B. anthracis produce potent toxins and are therefore a concern if used as biochemical war agents. C. botulinum produces neurotoxins that are among the most toxic known (10). In 2011, the Centers for Disease Control (CDC) announced that it was withdrawing provision of the investigational pentavalent botulinum toxoid vaccine that had been provided since 1965 for workers at risk from occupational exposure (11). This 30-year-old vaccine product was removed by the CDC as it had declined in immunogenicity and caused adverse effects at the injection site (11). However, more recently, the development of recombinant vaccines for botulism has focused on the nontoxic carboxyl terminus of the heavy-chain domain of C. botulinum serotypes A and B (12). B. anthracis toxins consist of a PA and two enzymes named edema factor and lethal factor. While a vaccine does currently exist for anthrax, it is far from ideal because it is administered in several doses, requires an adjuvant, and causes significant side effects (13). The main component for this vaccine is PA, and the vaccine is administered in 5 doses over a 12-month period and yearly thereafter. In previous work, a recombinant strain of L. acidophilus with plasmid expression of the PA was successful in protecting mice from infection in murine models (4).

In this study, we successfully expressed both the nontoxic host receptor-binding domain of the heavy chain of Clostridium botulinum serotype A neurotoxin (BoNT/A-Hc) and PA individually and together from the chromosome of L. acidophilus. The combination of chromosome-based expression and ease of oral administration and adjuvant attributes of L. acidophilus makes these recombinant strains promising candidates for the development of mucosal vaccines against botulism and anthrax.

## MATERIALS AND METHODS

### Bacterial strains and growth conditions.

Details of the bacterial strains and plasmids used in this study are provided in Table 1. L. acidophilus strains were propagated statically under ambient atmospheric conditions in de Man, Rogosa and Sharpe (MRS) broth (Difco Laboratories, Inc., Detroit, MI) or glucose semidefined medium (GSDM) (14) at 37°C or at 42°C when indicated. Escherichia coli strains were grown in brain heart infusion (Difco) broth at 37°C with aeration. Solid media contained 1.5% (wt/vol) bacteriological agar (Difco). Relevant antibiotics were included in the media when required (Table 1).

**TABLE 1:** Details of bacterial strains and plasmids used in this study

Strain or plasmid	Details	Antibiotic^*a*^ (μg/ml) used for selection	Reference
E. coli strain harboring synthetic construct			
NCK2225	Host for pTRK1100	Amp (50)	This study
Strains for plasmid-based expression			
E. coli strains			
MC1061	Transformation host for pTRK882-based plasmids	None	35
NCK1814	Host for pTRK882	Em (150)	36
NCK2062	Host for pTRK994	Em (150)	30
NCK2289	Host for pTRK1074	Em (150)	This study
L. acidophilus strains			
NCK1839	Host for pTRK896	Em (5)	4
NCK1895	Host for pTRK882	Em (5)	36
NCK2307	Host for pTRK1074	Em (5)	This study
			
Strains for chromosome-based expression			
E. coli strains			
EC101	RepA^+^ JM101; *repA* from pWV01 integrated in chromosome; cloning host for pORI-based plasmids	Kn (40)	37
EC1000	RepA^+^ MC1000, *repA* from pWV01 integrated in chromosome; cloning host for pORI-based plasmids	Kn (40)	38
NCK2169	Host for pTRK1038	Em (150), Kn (40)	9
NCK2309	Host for pTRK1078	Em (150), Kn (40)	This study
NCK2327	Host for pTRK1086	Em (150), Kn (40)	This study
NCK2343	Host for pTRK1088	Em (150)	This study
NCK2344	Host for pTRK1089	Em (150)	This study
L. acidophilus strains			
NCK1909	Δ*upp* (control strain; background strains for *upp*-based counterselective gene replacement)	None	19
NCK1910	NCK1909 harboring pTRK669; host for pORI-based counterselective integration plasmids	Cm (5)	19
NCK2310	BoNT/A-HcDCpep chromosomally integrated and expressed from the *lba0889* promoter	None	This study
NCK2326	PA-DCpep chromosomally integrated and expressed from the *lba0889* promoter	None	This study
NCK2345	PA-DCpep and BoNT/A-HcDCpep chromosomally integrated and expressed from the *lba0889* promoter	None	This study
Plasmid harboring synthetic vaccine cassettes			
pTRK1100	Source of codon-optimized BoNT/A-HcDCpep cassette	Amp	This study
			
Plasmids for plasmid-based expression			
pTRK882	Expression vector with *pgm* promoter	Em	36
pTRK896	pTRK882::PA-DCpep	Em	4
pTRK994	Expression vector with PA-DCpep	Em	30
pTRK1074	pTRK882::BoNT/A-HcDCpep cassette	Em	This study
Plasmids for construction of chromosome-based expression			
pTRK935	Counterselective integration vector with a *upp* expression cassette	Em	19
pTRK1038	Plasmid for targeted integration downstream of *lba0889*	Em	9
pTRK1078	pTRK1038::BoNT/A-HcDCpep cassette	Em	This study
pTRK1086	pTRK1038::PA-DCpep cassette	Em	This study
pTRK1088	Plasmid for targeted integration downstream of PA-DCpep in NCK2326	Em	This study
pTRK1089	pTRK1088::BoNT/A-HcDCpep cassette	Em	This study

a Amp, ampicillin; Em, erythromycin; Kn, kanamycin; Cm, chloramphenicol.

### DNA manipulations and sequence analysis.

Standard procedures were used for molecular manipulations (15). Genomic DNA was isolated using the ZR Fungal/Bacterial DNA miniprep kit (Zymo Research Corp., Orange, CA). Plasmid DNA from E. coli was isolated using the QIAprep Spin Miniprep kit (Qiagen Inc., Valencia, CA). Plasmid DNA was isolated from L. acidophilus strains using the QIAprep Spin Miniprep kit (Qiagen) with the following modifications. Briefly, the cell pellet was resuspended in P1 buffer containing 3 μl of a 10-U/μl stock solution of mutanolysin (Sigma-Aldrich, St. Louis, MO) and 5 mg/ml of lysozyme (Sigma-Aldrich) and incubated at 37°C for 20 min before proceeding with the remainder of the protocol. Chemically competent E. coli cells were prepared as described previously (16), and electrocompetent L. acidophilus cells were prepared as described by Walker et al. (17). Restriction enzymes (Roche Molecular Biochemicals, Indianapolis, IN) and T4 DNA ligase (New England BioLabs, Beverly, MA) were used according to the manufacturers' instructions. PfuUltra II Fusion HS DNA polymerase (Agilent, Santa Clara, CA) and the Expand High Fidelity PCR system (Roche) were used for cloning purposes, and Choice-*Taq* Blue DNA polymerase (Denville Scientific Inc., Metuchen, NJ) was used for PCR screening of transformants and recombinants. PCR products were purified using the QIAquick gel extraction kit (Qiagen). PCR primers (Table 2) were synthesized by Integrated DNA Technologies (Coralville, IA), and DNA sequencing was performed by Davis Sequencing, Inc. (Davis, CA), to confirm sequence fidelity.

**TABLE 2:** Details of PCR primers used in this study

Primer use and name	Primer sequence (5′ to 3′)^*a*^	Restriction site	Reference or source
Plasmid-based expression			
HcCAS_882F	AACTGCAGAGGAGAACGTATATGAAGAAG	PstI	This study
HcCASpep_882R	GGGGATCCTCATGGACGTTGTGGAGTTG	BamHI	This study
882_seqF	CTAATCGATGCATGCCATGGTGC	None	This study
882_seqR	GGTCGACAAGCAGGTATTCAG	None	This study
Chromosome-based expression			
BOT_upp_F1	ATAAGATTGCGGCCGCAGG	NotI	This study
BOT_upp_R1	AGATAGTTTA**GCGGCCGC**TCA	NotI	This study
SeqBot_1	CACCAGCTTGTGAAGCGTTA	None	This study
SeqBot_2	ACGGTGAGTATCACGACAAC	None	This study
SeqBot_3	GGGTCCACGTGGTTCAGTTA	None	This study
upp_Screen_F	GGGCTGGCTTAACTATGC	None	This study
upp_Screen_R	CTTCCGGCTCGTATGTTG	None	This study
PA_upp_F	GATC**GCGGCCGC**CAAAAAGGAGAACGTATATG	NotI	This study
PA_upp_R	GATC**GCGGCCGC**TTATGGTCTTTGTGGTGTAC	NotI	This study
SeqPA_1	AATACCGCTGATACAGCAAG	None	This study
MV_1	GATC**GGATCC**CGACTAAACCGGATATGACATT	BamHI	This study
MV_2	**GCGGCCGC**TTATGGTCTTTGTGGTGTAC	NotI	This study
MV_3	GTACACCACAAAGACCATAA**GCGGCCGC**ACCGAGTACAATTTCAACA	NotI	This study
MV_4	GATC**GAGCTC**CGCTATCAAGTAACTATTTATTAATTAATACTGGTG	SacI	This study
repA-F	TTGGGCGTATCTATGGCTGT	None	Goh and Klaenhammer, unpublished data
repA-R	CTGATAATTGCCCTCAAACCA	None	Goh and Klaenhammer, unpublished data
889screenF	GTTGTTGTTCCTGTAGGTAAGATTG	None	9
889screenR	CCACACTTTGAAGAAGGTTCTTG	None	9

a Restriction sites are shown in boldface.

### Codon optimization and gene synthesis of the BoNT/A-HcDCpep cassette.

The BoNT/A-HcDCpep vaccine cassette was composed of the amino acid sequence of the signal peptide from SlpA of L. acidophilus NCFM and BoNT/A-Hc with the DCpep (FYPSYHSTPQRP). This vaccine cassette was codon optimized for Lactobacillus gasseri ATCC 33323 using the Java Codon Adaptation Tool (JCat; http://www.jcat.de [18]). Restriction endonuclease sites, a ribosome binding site and spacer, and stop and start codons were added to the resulting codon-optimized sequence. The cassette was synthesized and cloned into pUC19minusMCS by Blue Heron Biotech (Bothell, WA), resulting in plasmid pTRK1100 (Table 1). This plasmid was used as a template for subsequent cloning.

### Construction of pTRK882-based expression plasmids.

The BoNT/A-HcDCpep vaccine cassette was amplified from pTRK1100 using primer pair HcCAS_882F and HcCASpep_882R (BoNT/A-HcDCpep) for construction of pTRK882-based expression plasmids (see Table S1 in the supplemental material). The resulting amplicons were ligated to the PstI and BamHI restriction sites of pTRK882, and ligation mixes were transformed into E. coli MC1061. Primer pair 882_seqF and 882_seqR was used to screen for transformants and to confirm insert integrity by DNA sequencing (Table 2). The resulting expression plasmid was designated pTRK1074 (BoNT/A-HcDCpep) (Table 1). The plasmid expressing the PA (pTRK896) was previously constructed (4) (Table 1).

### Integration of antigens into the L. acidophilus chromosome. (i) Chromosomal integration of the BoNT/A-Hc cassette.

For the construction of pTRK1038-based integration plasmids, primers BOT_upp_F1 and BOT_upp_R1 were used to amplify the vaccine cassette from pTRK1100, which was then cloned into the NotI site of pTRK1038 and transformed into E. coli EC101 (Table 1). Primer pair upp_Screen_F and upp_Screen_R was used to screen for transformants and to confirm insert integrity by DNA sequencing. The resulting integrative plasmid was designated pTRK1078 (BoNT/A-HcDCpep) (Table 1). Plasmid pTRK1078 was then used to integrate BoNT/A-HcDCpep into the L. acidophilus chromosome in the intergenic region between *lba0889* and *lba0888*. The methods used were described previously using the *upp*-based counterselectable gene replacement system (8, 19). Briefly, plasmid pTRK1078 was electroporated into NCK1910 (Table 1). NCK1910 harbors the helper plasmid pTRK669, which provides *repA* in *trans* for the replication of pTRK1078 (a pORI-based plasmid) (19). Procedures to isolate plasmid-free recombinants after targeted plasmid integration into the chromosome and double-crossover recombination were performed as described previously (19). Plasmid integrants were selected after propagating the transformants at 42°C for 30 generations in MRS containing 2 μg/ml of erythromycin. Screening with primer pair repA-F and repA-R confirmed the loss of pTRK669. Growth at 37°C for 30 generations without antibiotic pressure facilitated excision of the integrated plasmid backbone from the chromosome. Double-crossover recombinants were selected on GSDM containing 100 μg/ml of 5-fluorouracil, and BoNT/A-HcDCpep integrants were identified by PCR and verified by DNA sequencing using primer pair 889screenF and 889screenR (Table 2). The resulting strain expressing the BoNT/A-Hc cassette from the L. acidophilus chromosome was designated NCK2310 (Table 1).

### (ii) Chromosomal integration of the PA cassette.

Integration of PA-DCpep was performed as described above for the BoNT/A-Hc cassette. Primer pair PA_upp_F and PA_upp_R (Table 2) was used to amplify the PA from pTRK994 (Table 1). The amplicon was then cloned into the NotI site of pTRK1038 and transformed into EC1000 (Table 1). Primer pair upp_Screen_F and upp_Screen_R was used to screen for transformants and to confirm insert integrity by DNA sequencing. The resulting integrative plasmid was designated pTRK1086 (PA-DCpep) (Table 1). pTRK1086 was electroporated into NCK1910 (Table 1), and the remaining steps were performed as described above, resulting in the PA-DCpep chromosomal integration strain NCK2326.

### (iii) Construction of a multivalent vaccine.

To facilitate insertion of the BoNT/A-Hc cassette downstream of PA-DCpep in NCK2326, first the 709-bp and 735-bp flanking regions of the intergenic region between the PA-DCpep and *lba0888* in NCK2326 were amplified with primer pairs MV_1/MV_2 and MV_3/MV_4, respectively (Table 2). These amplicons were purified and joined by splicing using overlap extension PCR, digested with SacI and BamHI, and subsequently ligated into the SacI and BamHI sites of pTRK935 (Table 1). Ligation mixtures were transformed into E. coli EC101, and constructs were confirmed by sequencing. The resulting integrative plasmid was designated pTRK1088 (Table 1). Subsequently, the BoNT/A-Hc cassette was amplified using primers BOT_upp_F1 and BOT_upp_R1 from pTRK1100, and the purified amplicon was then cloned into the NotI site of pTRK1088 and transformed into E. coli EC101 (Table 1). Primer pair upp_Screen_F and upp_Screen_R was used to screen for transformants. In addition to primer pair upp_Screen_F and upp_Screen_R, primers SeqBot_1, SeqBot_2, and SeqBot_3 were used to confirm insert integrity by DNA sequencing. The resulting integrative plasmid was designated pTRK1089 (Table 1). Plasmid pTRK1089 was transformed into L. acidophilus NCK2326 containing pTRK669 (to provide *repA* in *trans* for replication of pTRK1089). The presence of pTRK669 was confirmed using plasmid-specific primers repA-F and repA-R (Table 2). Procedures to isolate plasmid-free recombinants after targeted plasmid integration into the chromosome and double-crossover recombination were performed as described above and previously (19). The primer pair 889screenF and SeqPA_1, in addition to sequencing primers SeqBot_2, SeqBot_3, and PA_upp_R (Table 2), was used to confirm insertion of BoNT/A-Hc downstream of the PA cassette in the chromosome. The resulting multivalent strain with the PA and the BoNT/A-Hc cassette integrated downstream of *lba0889* was designated NCK2345.

### Stability assays and growth curves.

L. acidophilus plasmid-based strains (NCK1839 and NCK2307, with antibiotic selection) and integrant recombinant strains (NCK2310, NCK2326, and NCK2345, without antibiotic selection) were grown in MRS broth overnight. Subsequently, all strains were grown in duplicate in MRS without antibiotic selection and transferred 14 times (1% inoculum). NCK1839 and NCK2307 were serially diluted and plated (days 1, 3, 6, 9, 11, and 14) on MRS agar with and without Em selection to determine the plasmid stability of pTRK896 and pTRK1074. Plasmid stability was determined as the percentage of CFU resistant to erythromycin (Em). PCR was used to confirm the presence or absence of the plasmid from NCK1839 and NCK2307 and of the vaccine cassettes from NCK2310, NCK2326, and NCK2345 from 20 colonies after seven transfers.

Growth experiments with the integrants NCK2310, NCK2326, and NCK2345 and control strain NCK1909 were done in MRS broth to stationary phase, and the strains were subsequently inoculated (1%) into MRS broth and grown for 25 h at 37°C. Measurements of optical density at 570 nm (OD_570_) were recorded every hour over a 25-h period using a microtiter plate reader (FLOUStar Optima; BMG Technologies, Durham, NC). In addition, after 16 h of growth in MRS broth, cells were enumerated by plating on MRS agar plates.

### RNA isolation.

Total RNA was isolated from the L. acidophilus integration strains (NCK2310, NCK2326, and NCK2345) grown to mid-log phase (OD_600_ ∼ 0.6). RNA isolated from mid-log-phase cells of the control strain NCK1909 and subsequent RNA sequencing data (RNA-seq) were used from a previous study (Yong Jun Goh, unpublished data). Cells were harvested by centrifugation (1,717 × *g* for 10 min at room temperature [RT]), and total RNA was extracted using Tri-Reagent (Life Technologies, Carlsbad, CA) and purified with the RNeasy minikit (Qiagen). Briefly, the cell pellets from the 10-ml cultures were resuspended in 1 ml of Tri-Reagent, added to a screw-cap tube containing beads (0.1-mm glass beads; Bio-Spec) and bead beaten for 5 min (5 times each for 1-min intervals with 1 min on ice after each interval). Subsequently, 200 μl of chloroform was added to each sample. Samples were then left on ice for 20 min and centrifuged at 16,873 × *g* for 20 min at 4°C. The aqueous layer was transferred to a fresh tube, and RNA was subsequently purified with the RNeasy minikit (Qiagen) using the cleanup procedure according to the manufacturer's instructions. RNA integrity was checked by gel electrophoresis. DNA was removed by incubating samples with Turbo DNase as described by the manufacturer (Ambion Inc., Austin, TX), purified using the RNeasy minikit (Qiagen), and checked for integrity by gel electrophoresis. Samples were confirmed to be DNA free by PCR with L. acidophilus NCFM gene-specific primers. RNA integrity was confirmed by capillary electrophoresis on the Agilent Bioanalyzer (Agilent Technologies, Santa Clara, CA).

### RNA sequence transcriptional analysis.

The High-Throughput Sequencing and Genotyping Unit of the Roy J. Carver Biotechnology Center, University of Illinois at Urbana-Champaign, performed the preparation of the mRNA libraries and RNA sequencing. Initially, rRNA was removed with the Ribozero Bacteria kit (Illumina, San Diego, CA) followed by library preparation with the TruSeq Stranded RNA Sample Prep kit (Illumina, CA). The libraries were pooled in equimolar concentration, and each pool was quantitated by quantitative PCR (qPCR) and sequenced on one lane for 161 cycles using the Illumina HiSeq 2500 Ultra-High-Throughput Sequencing system (with a read length of 160 nucleotides) with the HiSeq SBS sequencing kit version 4. Fastq files were generated and demultiplexed with the bcl2fastq v2.17.1.14 Conversion Software (Illumina). Adapter sequences were removed, and raw sequences were assessed for quality using Fast QC version 0.11.4 (http://www.bioinformatics.babraham.ac.uk/projects/fastqc/). Subsequent processes were performed with Geneious 9.0.5 (20). Raw reads were trimmed to remove bases with an error probability limit of 0.001 (Phred score of 30), and reads of <20 nucleotides were removed. These sequences were then mapped to the reference genomes (accession numbers NC_006814 and modified NC_006814 for strains NCK2310, NCK2326, and NCK2345) using the Geneious mapper (20). Geneious software was used to calculate the normalized transcripts per million (TPM) and to compare expression levels between the control (NCK1909) and integration (NCK2310, NCK2326, and NCK2345) strains. For this study, genes were considered differentially expressed if they had a log_2_ differential expression value greater than 2 and less than −2 and a differential expression absolute confidence (the negative base 10 log of the *P* value) value greater than 6.

### Western blot assays.

L. acidophilus plasmid-based strains (NCK1839, NCK1895, and NCK2307 with antibiotic selection), integrant recombinant strains (NCK2310, NCK2326, and NCK2345, without antibiotic selection), and the control strain (NCK1909, without antibiotic selection) were grown in MRS broth overnight. These cultures were used for inoculation at 1% (vol/vol) into 10 ml of GSDM and propagated at 37°C to stationary phase (16 h). Erythromycin (5 μg/ml) was included in the media for plasmid-based recombinant strains (Table 1). Cells were harvested by centrifugation (1,717 × *g* for 10 min at room temperature), and culture supernatants (10 ml) were concentrated. Concentrated cell-free supernatants were prepared by passing the 10-ml supernatant through Amicon Ultra columns (30-kDa molecular mass cutoff; EMD Millipore Corporation, Billerica, MA) by centrifugation according to the manufacturer's instructions with a final wash using 4 ml of phosphate-buffered saline (PBS; pH 7.4; Life Technologies, Carlsbad, CA). The concentrate was removed to a fresh tube, and the filter column was washed with 50 μl of PBS, which was then added to the concentrate. For preparation of cell lysates, the cell pellets from the 10-ml cultures were each resuspended in 1 ml of PBS, transferred to a screw-cap tube containing beads (0.1-mm glass beads; Bio-Spec, Bartlesville, OK), and bead beaten for 5 min (5 times each for 1-min intervals with 1 min on ice after each interval). Bradford reagent (Sigma-Aldrich) was used to determine the protein concentrations in the concentrated cell-free supernatants and cell lysates according to the manufacturer's instructions. Prior to Western blot assays, protein concentrations were standardized for the cell lysate and cell-free supernatant samples in PBS and boiled for 10 min with Laemmli sample buffer. Protein samples were loaded onto 4 to 15% Mini-Protean TGX gels (Tris-Glycine eXtended; Bio-Rad, Hercules, CA). NCK1895 and NCK1909 served as negative controls, and rBoNT/A-Hc (53.5 kDa) and rPA (83 kDa) were used as positive controls. Gels were transferred to polyvinylidene difluoride (PVDF) membranes using the Turbo Blot Turbo transfer system with the Trans-Blot Turbo transfer pack (Bio-Rad) according to the manufacturer's instructions. Membranes were blocked for 1 h with 10 ml of 5% nonfat dried milk (NFDM) in Tris-buffered saline with Tween 20 (TBST). Primary antibody was then added in 10 ml of 5% NFDM in TBST (C. botulinum neurotoxin type A specific IgG, rabbit, 1:2,700; Metabiologics Inc., Madison, WI; for PA, anti-protective antigen from B. anthracis, goat, 1:5,000; List Biological Laboratories, Campbell, CA), and the mixture was incubated overnight at 4°C. Membranes were washed 4 times with TBST (10 ml for 5 min, with shaking). Subsequently, secondary antibody (for BoNT/A, goat anti-rabbit, 1:3,000; for PA, rabbit anti-goat, 1:5,000; both from Bio-Rad) was added in 10 ml of 5% NFDM in TBST and left shaking at room temperature for 1 h. Membranes were washed 4 times with TBST (10 ml for 5 min) and once with Tris-buffered saline (10 ml for 5 min) and then treated with the Clarity Western ECL Substrate (Bio-Rad) according to the manufacturer's instructions.

## RESULTS

### Design and codon optimization of the BoNT vaccine cassette.

**FIG 1:**
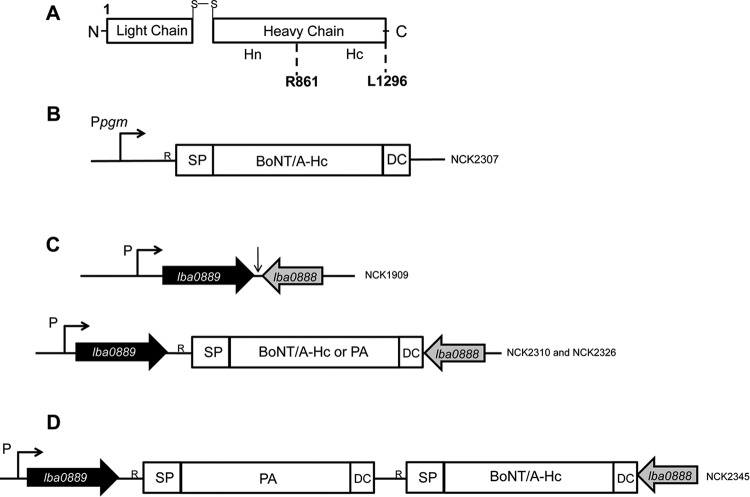
Schematic of the BoNT/A neurotoxin and vaccine cassettes. (A) The Hc region (nontoxic host receptor-binding domain) is shown from amino acid positions R861 to L1296. N and C represent the N and C termini, respectively. Hn, translocation domain. (B) Schematic of BoNT/A vaccine cassette under expression control of the *pgm* (P*pgm*) promoter in pTRK882. R, ribosome binding site. SP, signal peptide. BoNT/A-Hc, antigen. DC, dendritic-cell-targeting peptide. (C) Schematic of the chromosomal region of L. acidophilus targeted for integration with the BoNT/A or PA vaccine cassette (the black vertical arrow indicates the targeted region). (D) Schematic of the multivalent strain expressing the BoNT/A and PA vaccine cassettes. Note that *lba0889* is carried on the antisense strand but is shown here as 5′ to 3′.

C. botulinum neurotoxins are 150-kDa proteins comprising a 50-kDa light chain and a 100-kDa heavy chain linked by a disulfide bond (12) (Fig. 1A). The light chain functions in the cleavage of the eukaryotic synaptosome-associated protein 25 (SNAP-25), whereas the heavy chain is composed of a translocation (Hn) and a host receptor-binding domain (Hc) (Fig. 1A). The nontoxic host receptor binding domain has been targeted for research on recombinant vaccines. Initial work with heterologous expression of the native BoNT/A sequence in E. coli was unsuccessful due to instability of the native coding sequence. Subsequent codon optimization of the coding sequence resulted in successful expression both in E. coli and later in Pichia pastoris (21, 22). Therefore, in our study, we codon optimized the BoNT/A vaccine cassette using the online JCat tool for expression in lactobacilli. The codon-optimized BoNT/A-Hc nucleotide sequence resulted in 75% sequence homology with the native BoNT/A-Hc sequence (GenBank accession number X52066). The vaccine cassette was composed of the amino acid sequence of the signal peptide from SlpA of L. acidophilus NCFM (GenBank accession number AAV42070), the BoNT/A-Hc amino acid sequence (GenBank accession number WP_011948511.1 amino acid position from R861 to L1296, 436 amino acids), and the DC-targeting peptide (FYPSYHSTPQRP) (5) (Fig. 1B). Subsequently, the vaccine cassette (481 amino acids, 55.4 kDa) was synthesized and cloned into the pUC19minusMCS vector. The resulting plasmid pTRK1100 was then used as a template for PCR amplification of the vaccine cassette and subsequent cloning into pTRK882 and pTRK1038 (Table 1).

### Expression of antigens from pTRK882.

Plasmid pTRK882 contains a constitutively strong *pgm* (phosphoglycerate mutase) promoter and has been used previously for the expression of anthrax PA with the DC peptide (L. acidophilus NCK1839 harboring plasmid pTRK896 (Table 1) (4). In this study, the BoNT/A-Hc-DCpep cassette was cloned into pTRK882 (Fig. 1B), and the resulting plasmid was designated pTRK1074 (Table 1). Plasmid pTRK1074 was then transformed into L. acidophilus NCFM. The recombinant strain was designated NCK2307 (BoNT/A-Hc-DCpep) (Table 1). For clarity, from this point forward, the BoNT/A-Hc-DCpep cassette is referred to as BoNT/A and the PA-DCpep cassette as PA.

### Construction of chromosomal integration strains. (i) Directed chromosomal integration of BoNT/A and PA cassettes in L. acidophilus.

**FIG 2:**
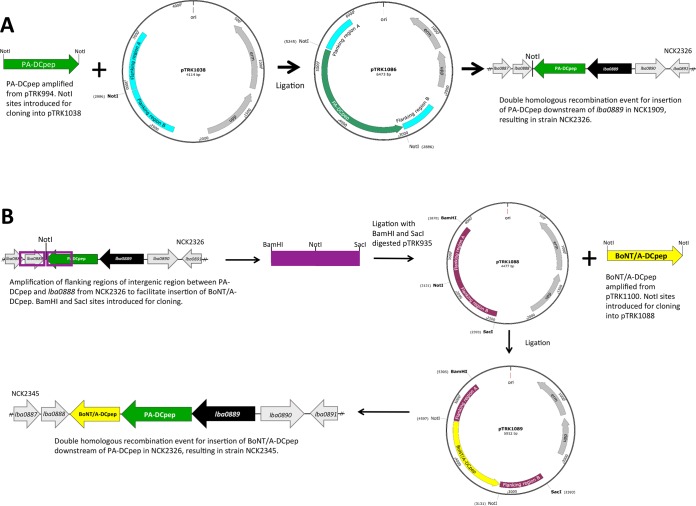
Construction of the multivalent strain L. acidophilus NCK2345. (A) The PA-DCpep was amplified from pTRK994 and cloned into the NotI site of pTRK1038. The *upp*-based counterselective gene replacement system in conjunction with pTRK1086 was used to construct L. acidophilus expressing PA-DCpep (NCK2326). (B) To construct the multivalent strain, flanking regions of the intergenic region between the PA-DCpep and *lba0888* were amplified (including the NotI site introduced as described for panel A) and cloned into pTRK935, resulting in pTRK1088. The BoNT/A-DCpep was subsequently cloned into the NotI site of pTRK1088, resulting in pTRK1089. The *upp*-based counterselective gene replacement system in conjunction with pTRK1089 was used to construct L. acidophilus expressing PA-DCpep and BoNT/A-DCpep (NCK2345).

Plasmid pTRK1038 contains flanking regions of the intergenic region between genes *lba0889* and *lba0888* from L. acidophilus (denoted by the vertical arrow in Fig. 1C). This facilitates insertion of the cassettes under the transcriptional control of the strong native *lba0889* promoter in the chromosome (9). The BoNT/A cassette was cloned into the NotI site of the integration vector pTRK1038, and the resulting plasmid was designated pTRK1078 (Table 1). The *upp*-based counterselective gene replacement system for L. acidophilus (19) and pTRK1078 were subsequently used to integrate the BoNT/A cassette into the chromosome of L. acidophilus (Fig. 1C). The resulting integrant strain was designated NCK2310 (BoNT/A) (Table 1). The same strategy was employed to clone the PA into the NotI site of the integration vector pTRK1038, resulting in plasmid pTRK1086 (Fig. 2A; Table 1). Plasmid pTRK1086 was subsequently transformed into strain NCK1910, and by following the procedures detailed above, the PA was successfully integrated into the intergenic region between genes *lba0888* and *lba0889*, resulting in strain NCK2326 (PA) (Fig. 2A).

### (ii) Construction of a multivalent vaccine.

L. acidophilus NCK2326 was then used as the background strain for construction of the multivalent strain (Fig. 1D). First, the helper plasmid pTRK669 was transformed into NCK2326, providing *repA* in *trans*. Second, a plasmid that would facilitate insertion downstream of the PA in NCK2326 was constructed. This plasmid (pTRK1088) was constructed by amplifying the flanking regions of the intergenic region between the PA and *lba0888* from NCK2326 (Fig. 2B). These flanking regions included the NotI site that was introduced into the chromosome from the construction of strain NCK2326. This NotI site facilitated cloning of the BoNT/A cassette into pTRK1088 (Fig. 2B). The BoNT/A was amplified from pTRK1100 and cloned into the NotI site of pTRK1088, resulting in plasmid pTRK1089 (Table 1). This plasmid was then successfully transformed into NCK2326 containing pTRK669. Using the *upp*-based counterselective gene replacement system for L. acidophilus (19) and pTRK1089, the BoNT/A cassette was successfully integrated into the chromosome of NCK2326 downstream of the PA cassette (Fig. 2A), resulting in strain NCK2345 (PA and BoNT/A) (Fig. 2B).

### Stability assays.

Plasmid stability assays showed loss of the antigen expression plasmids, demonstrating the need for stable chromosomal expression. Both plasmid- and integration-based strains were cultivated in antibiotic-free medium for 14 days. By day 3, only 12% of L. acidophilus NCK2307 (BoNT/A) and 24% of NCK1839 (PA) maintained erythromycin resistance (see Fig. S1A in the supplemental material). By day 6, these numbers had dropped to 1% and 3%, respectively, and to less than 1% for NCK2307 and NCK1839 by day 14 (see Fig. S1A in the supplemental material). PCR on colonies after 7 transfers confirmed the presence of the insert in the three integration strains from 100% of tested colonies, whereas both plasmids pTRK896 (PA) and pTRK1074 (BoNT/A) were retained in only 5% of the tested colonies (data not shown).

### RNA sequencing and transcriptional analysis.

**FIG 3:**
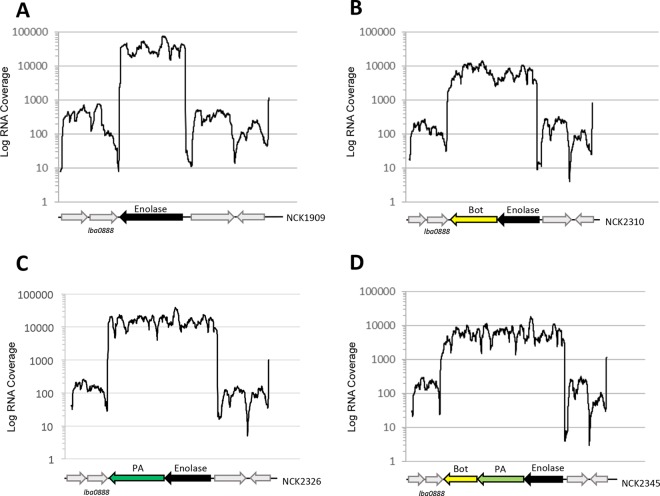
Transcriptional profiles of genes surrounding *lba0889* from control and integration strains. RNA-seq revealed transcription profiles for the control strain (NCK1909) (A), the integration expressing BoNT/A (NCK2310) (B), the integration expressing PA (NCK2326) (C), and the multivalent strain expressing BoNT/A and PA (NCK2345) (D). A black arrow indicates the enolase (LBA0889), a yellow arrow indicates the BoNT/A, a green arrow indicates the PA, and the light gray arrows indicate the surrounding hypothetical genes.

Total RNA from the integration strains NCK2310 (BoNT/A), NCK2326 (PA), and NCK2345 (BoNT/A and PA) was isolated and sent for RNA-seq. Transcriptional analysis through RNA-seq confirmed that the enolase gene (*lba0889*) is highly expressed (Fig. 3A). Based on TPM values, *lba0889* was the seventh-most highly expressed gene in the genome of the control strain L. acidophilus NCK1909. The enolase was also confirmed to be monocistronic. Transcriptional data indicated high expression of BoNT/A (Fig. 3B), PA (Fig. 3C), and both antigens in the multivalent strain (Fig. 3D) downstream of *lba0889*, demonstrating that expression of these antigens was successfully driven by the enolase promoter.

**FIG 4:**
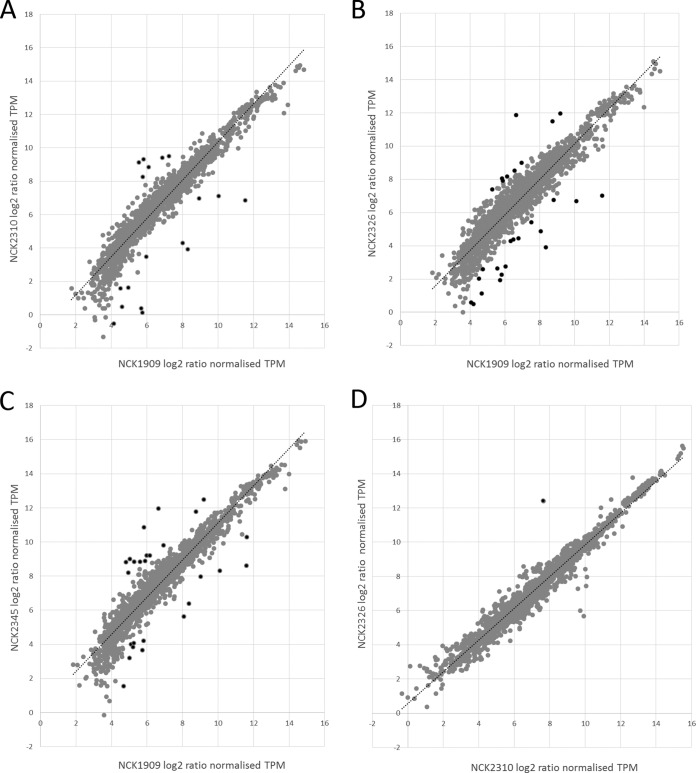
Pairwise comparisons of whole-genome transcriptional profiles of the chromosomal integrants. Plots of log_2_ transformed normalized transcripts per million of open reading frames from NCK1909 (control) versus NCK2310 (BoNT/A) (A), NCK1909 (control) versus NCK2326 (PA) (B), NCK1909 (control) versus NCK2345 (BoNT/A and PA) (C), and NCK2310 (BoNT/A) versus NCK2326 (PA) (D). Data points in black represent differentially expressed genes in each pairwise comparison and are detailed in Table S1 in the supplemental material.

Pairwise comparisons of whole-transcriptome data were also performed between the control strain (NCK1909) and each of the three integration strains to determine the effects of heterologous expression of the antigens on the rest of the transcriptome. Data indicated minimal differences between the control and integration strains (Fig. 4). In fact, transcription profiles of the surrounding genes were very similar for all four strains (Fig. 3A to D). The total genes differentially expressed between the control strain and the BoNT/A integration, PA integration, and BoNT/A and PA multivalent strains were 0.97, 1.42, and 1.32%, respectively. The majority of differentially expressed genes were hypothetical proteins with unknown functions (see Table S1 in the supplemental material). *lba0888* was differently expressed in the integrant strains that contained the PA but not the BoNT/A antigen (see Table S1 in the supplemental material). The gene encoding LBA0888 is located upstream on the forward strand (Fig. 3) of the enolase gene (*lba0889*, on the reverse strand), which encodes an uncharacterized protein with a conserved 140-amino-acid bacterial domain of unknown function (DUF4767, IPR031927). It has no signal peptide and has low amino acid identity (∼50% or less) to orthologs in other lactobacilli, whereas it is conserved in the available sequenced L. acidophilus strains. *lba0888* was the only differentially expressed gene when the whole transcriptome was compared between NCK2310 (BoNT/A) and NCK2326 (PA) (Fig. 4D; see also Table S1 in the supplemental material). Growth curves indicated no difference in growth of the three integration strains with the vaccine cassettes compared to the control strain (see Fig. S1B in the supplemental material). Cell counts for NCK1909, NCK2310, NCK2326, and NCK2345 were 2.48 × 10^8^ ± 3.68 × 10^7^, 2.04 × 10^8^ ± 5.66 × 10^6^, 2.90 × 10^8^ ± 5.66 × 10^6^, and 2.51 × 10^8^ ± 1.56 × 10^7^, respectively, after 16 h of growth in MRS broth.

### Confirmed expression of BoNT/A and PA by Western blotting assays.

**FIG 5:**
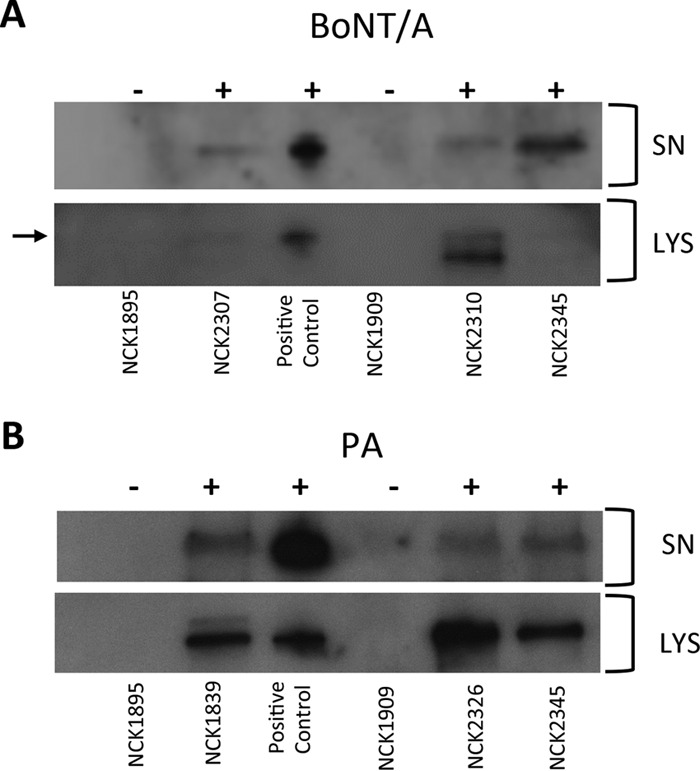
Detection of BoNT/A and PA expression by Western blot assays. Western blot assays showed detection of BoNT/A (A) and PA (B) in both concentrated cell-free supernatants (SN) and cell lysates (LYS). Positive controls were rBoNT/A-Hc (53.5 kDa) and rPA (83 kDa). The arrow in panel A indicates the mature BoNT/A. L. acidophilus NCK1895 containing the empty plasmid pTRK882 was used as a negative control for the plasmid-based strains (NCK2307, BoNT/A; NCK1839, PA). L. acidophilus NCK1909 was used as the negative control for the integration strains: NCK2310 (BoNT/A), NCK2326 (PA), and NCK2345 (BoNT/A and PA).

To determine whether the recombinant strains expressed BoNT/A (NCK2310), PA (NCK2326), and both antigens (NCK2345), Western blot assays were performed. The plasmid-based strains expressing BoNT/A (NCK2307) and PA (NCK1839) were also included in Western blot assays, allowing comparison of chromosomal and plasmid-based expression of the antigens. L. acidophilus NCK1895, which harbors the empty vector pTRK882, was used as a control strain for the plasmid-based recombinants, and the parent strain, L. acidophilus NCK1909, was used as a control for the integrants (Table 1 and Fig. 5). Western blot assays confirmed expression of BoNT/A in cell lysates and cell-free supernatant samples from plasmid expression and the integrant and the multivalent strains (Fig. 5A). Expression of the PA was also detected from the plasmid, single integrant, and multivalent strains (Fig. 5B), confirming the results from the whole-genome transcriptome data (Fig. 3).

## DISCUSSION

In this study, we utilized L. acidophilus for mucosal delivery of BoNT/A-Hc and PA, either as a single-antigen or a multivalent vaccine. A multivalent vaccine is warranted for C. botulinum and B. anthracis because they both often top the list for bioterrorism threats. L. acidophilus is an ideal candidate for the delivery of antigens for mucosal immunity, as the route of delivery is particularly important in the case of antigen presentation against BoNTs and for inhaled anthrax. Previous research on BoNT adsorption demonstrated that antigen presentation via the mucosal route is preferred over an intramuscular injection (23–26). The availability and advances in genetic tools have facilitated functional analysis of numerous genes and groups of genes that contribute to the probiotic functionality of L. acidophilus (19, 27). More recently, these tools have been utilized to investigate the interaction of L. acidophilus with immune cells such as dendritic cells (3). Additionally, through deletion of specific genes such as the gene encoding a phosphoglycerol transferase involved in lipoteichoic acid biosynthesis, L. acidophilus was successfully targeted to applications such as treatment of intestinal inflammation and colon cancer polyposis (28, 29). Subsequently, recombinant strains of L. acidophilus have been constructed for vaccine delivery (4, 6, 7). Building on this body of work, high-expression regions of the chromosome were identified with a view to utilizing these regions for expression of antigens and mucosal vaccine delivery (9). This strategy negates the need for antibiotic selective pressure for plasmid maintenance and potential problems of plasmid instability. In the current study, we used this strategy to integrate BoNT/A and PA into the chromosome. Previous work has shown increased vaccine efficacy by the addition of a DC-targeting peptide at the 3′ end of the engineered vaccine cassette (4, 30). Therefore, we included this motif in our vaccine cassettes. The PA cassette also contains the DC-targeting peptide and its native signal peptide (4). The BoNT/A cassette was codon optimized for Lactobacillus and was successfully cloned in both our plasmid and chromosomal expression systems. Our previous studies showed that codon optimization was not required for expression of the PA in lactobacilli (4, 30). Our data demonstrated loss of the plasmid expressing both the BoNT/A and PA cassettes when antibiotic selection was removed. In fact, within 3 days over 70% of the cell population had lost the plasmids, emphasizing the importance of integrating the antigens into the chromosome. We used the well-established *upp*-based counterselective gene replacement system for L. acidophilus to precisely insert the BoNT/A and PA into the intergenic region downstream of *lba0889*. *lba0889*, which encodes an enolase, was predicted to be expressed in high numbers in the L. acidophilus transcriptome based on microarray analysis (9, 31). Our RNA sequence data confirmed that *lba0889* was monocistronic and one of the most highly expressed genes in the genome. This gene was also shown to be highly expressed in the murine gastrointestinal tract (Yong Jun Goh, personal communication) and in other species of lactobacilli (32). After insertion of the BoNT/A and PA cassettes individually or in tandem downstream of the enolase gene, high expression of the cassettes was observed across the newly formed operons. Western blot assays detected protein expression from both cell lysates and cell-free supernatants for all three vaccine integrants. In particular, these assays indicated that secretion of the BoNT/A varied between the multivalent strain and the strain in which the BoNT/A was expressed without the PA. The reason for this difference in the immunoblots is unknown. As these are late-stationary-phase cultures, we would expect that some of the antigens are released into the cell-free supernatant by cell lysis. To date, the majority of studies for expression of antigens in lactobacilli has relied on plasmids (1). A few studies have targeted lactobacilli for chromosomal expression of antigens by replacing native genes with the antigen of interest (33) and/or inserting a heterologous promoter (34). This study demonstrated that antigens of interest can be inserted into high-expression regions of the genome in a precise, targeted fashion without the need for external promoters or gene inactivation. RNA-seq was also used to determine that insertion of the heterologous vaccine cassettes had minimal effect on the whole transcriptome. In the case where the PA cassette was inserted either singularly or in tandem with the BoNT/A cassette, upregulation of the adjoining gene *lba0888* was observed. The function of LBA0888 is unknown; this protein is encoded by the strand opposite to the enolase gene (*lba0889*), suggesting that insertion of the PA cassette had a steric effect on transcription resulting in increased expression of *lba0888*. Growth curves over 25 h and cell counts per ml after 16 h of growth in MRS broth indicated no difference in growth between the control strain and the three integration strains, demonstrating that the transcriptional changes observed had no major effect on cell growth under standard laboratory conditions.

This study has demonstrated that heterologous BoNT/A and PA protein expression directed from a single high-expression region of the L. acidophilus chromosome is as efficient as a multicopy plasmid-based protein expression. Additionally, we can target further high-expression regions of the chromosome to facilitate the expression of multiple antigens from a single strain or multiple copies of single antigens. Chromosomally expressed antigens are more favorable, as undesired traits such as plasmid instability and the need for antibiotic maintenance are omitted. The combination of chromosomal expression in conjunction with the DC-targeting peptide in a LAB strain that survives passage through the gastrointestinal tract paves the way for future studies on efficient vaccine delivery.

## Supplementary Material

Supplemental material
