# Supplementary material for: Multivalent Chromosomal Expression of the Clostridium botulinum Serotype A Neurotoxin Heavy-Chain Antigen and the Bacillus anthracis Protective Antigen in Lactobacillus acidophilus
Source: Appl Environ Microbiol. 2016 Sep 30;82(20):6091–101. doi: 10.1128/AEM.01533-16 (PMC5068166; doi:10.1128/AEM.01533-16)
Supplement: Supplemental material [file supp_82_20_6091__index.html]

Supplemental material 

# Multivalent Chromosomal Expression of the Clostridium botulinum Serotype A Neurotoxin Heavy-Chain Antigen and the Bacillus anthracis Protective Antigen in Lactobacillus acidophilus

## Supplemental material

- Supplemental file 1 -

  Differentially expressed genes from pairwise comparisons shown in Fig. 4 (Table S1); plasmid stability assays and growth curves (Fig. S1).

  PDF, 445K
