# Supplementary material for: Multivalent Chromosomal Expression of the Clostridium botulinum Serotype A Neurotoxin Heavy-Chain Antigen and the Bacillus anthracis Protective Antigen in Lactobacillus acidophilus
Source: Appl Environ Microbiol. 2016 Sep 30;82(20):6091–101. doi: 10.1128/AEM.01533-16 (PMC5068166; doi:10.1128/AEM.01533-16)
Supplement: Supplemental material [file AEM.01533-16_zam999117452so1.pdf]

Multivalent chromosomal expression of the *Clostridium botulinum* serotype A neurotoxin heavy chain antigen and *Bacillus anthracis* protective antigen in *Lactobacillus acidophilus*

O'Flaherty and Klaenhammer

Supplementary Figures and Tables:

Table S1: Differentially expressed genes from pairwise comparisons shown in Fig. 4

Name	locus_tag	Differential Expression Ratio	Differential Expression Log2 Ratio	Differential Expression Absolute Confidence	Differential Expression p-value
**NCK2310 vs. NCK1909 (BoNT/A vs. Control)**		**NCK2310/ NCK1909**			
ABC transporter permease	LBA1679	11.9	3.6	1000	0.00E+00
ABC transporter ATP-binding protein	LBA1680	11.3	3.5	1000	0.00E+00
hypothetical protein	LBA1184	6.8	2.8	1000	0.00E+00
hypothetical protein	LBA1186	5.9	2.6	1000	0.00E+00
OppA	LBA1665	5.7	2.5	1000	0.00E+00
hypothetical protein	LBA1187	4.8	2.2	1000	0.00E+00
hypothetical protein	LBA0485	-3.9	-2	1000	0.00E+00
hypothetical protein	LBA1791	-5.6	-2.5	15.9	1.20E-16
hypothetical protein	LBA0486	-7.6	-2.9	1000	0.00E+00
hypothetical protein	LBA1072	-7.7	-2.9	6.9	1.40E-07
hypothetical protein	LBA1803	-7.8	-3	13.4	4.40E-14
hypothetical protein	LBA1789	-10	-3.3	10.1	7.10E-11
transposase	LBA1723	-13	-3.7	1000	0.00E+00
hypothetical protein	LBA0509	-17.1	-4.1	9.9	1.30E-10
hypothetical protein	LBA0490	-20.7	-4.4	1000	0.00E+00
hypothetical protein	LBA1756	-25.4	-4.7	1000	0
hypothetical protein	LBA0508	-27.5	-4.8	7.6	2.50E-08
hypothetical protein	LBA1801	-37.6	-5.2	22.4	3.90E-23
hypothetical protein	LBA1802	-50.1	-5.6	23.8	1.70E-24
**NCK2326 vs. NCK1909 (PA vs. Control)**		**NCK2326/ NCK1909**			
hypothetical protein	LBA0888	37.5	5.2	1000	0
DNA-damage-inducible protein J	LBA1565	6.8	2.8	1000	0
hypothetical protein	LBA1564	6.7	2.7	1000	0
aggregation promoting protein	LBA0493	4.6	2.2	1000	0
flavodoxin	LBA1563	4.3	2.1	1000	0
hypothetical protein	LBA0133	4.1	2	1000	0
ABC transporter ATP-binding protein/permease	LBA1276	4.1	2	1000	0
LysA CDS	LBA1918	4.1	2	1000	0
ABC transporter permease CDS	LBA0321	3.9	2	1000	0
PTS system cellobiose-specific transporter subunit IIC	LBA0876	-4.1	-2	1000	0

kanamycin kinase	LBA1348	-4.1	-2	8.1	7.9E-09
GlgC	LBA0681	-4.2	-2.1	17.8	1.7E-18
heavy-metal-transporting ATPase	LBA0542	-4.3	-2.1	40.4	4.2E-41
GlgB	LBA0680	-4.3	-2.1	20.2	6E-21
AckA	LBA1873	-4.4	-2.1	6.3	5.3E-07
hypothetical protein	LBA1797	-5	-2.3	27.4	3.7E-28
cysteine synthase	LBA1088	-5.5	-2.5	6.3	4.8E-07
hypothetical protein	LBA1803	-7.5	-2.9	15.3	4.9E-16
transposase	LBA1723	-9.1	-3.2	1000	0
hypothetical protein	LBA1791	-9.9	-3.3	23.1	8.9E-24
hypothetical protein	LBA0486	-10.6	-3.4	1000	0
HAD family hydrolase	LBA0564	-10.8	-3.4	6	0.0000009
hypothetical protein	LBA0509	-11.8	-3.6	9.7	2.1E-10
hypothetical protein	LBA1802	-11.9	-3.6	21	1.1E-21
hypothetical protein	LBA0508	-13.8	-3.8	6.9	1.2E-07
hypothetical protein	LBA1801	-13.9	-3.8	21.3	4.7E-22
hypothetical protein	LBA0490	-22	-4.5	1000	0
hypothetical protein	LBA1756	-23.6	-4.6	1000	0
**NCK2345 vs. NCK1909 (BoNT/A and PA vs. Control)**		**NCK2345/ NCK1909**			
hypothetical protein	LBA0888	20.9	4.4	1000	0
OppA	LBA1665	17.2	4.1	1000	0.00E+00
ABC transporter	LBA1044	8.5	3.1	1000	0.00E+00
glutamine ABC transporter ATP-binding protein	LBA1045	8.3	3.1	1000	0.00E+00
glutamine ABC transporter substrate-binding protein	LBA1046	6.3	2.6	1000	0.00E+00
DNA-damage-inducible protein J	LBA1565	5.1	2.4	1000	0
glutamine ABC transporter membrane spanning permease	LBA1042	5	2.3	1000	0.00E+00
ABC transporter permease	LBA1679	4.9	2.3	1000	0.00E+00
maltose phosphorylase	LBA1870	4.9	2.3	1000	0.00E+00
hypothetical protein	LBA1184	4.4	2.1	1000	0.00E+00
hypothetical protein	LBA1564	4.3	2.1	1000	0.00E+00
ABC transporter ATP-binding protein	LBA1680	4.2	2.1	1000	0.00E+00
hypothetical protein	LBA1186	3.9	2	1000	0.00E+00
hypothetical protein	LBA0485	-3.9	-2	1000	0.00E+00
hypothetical protein	LBA1694	-4.1	-2	6.6	2.80E-07
transcriptional regulator family protein	LBA1444	-4.4	-2.1	7.9	1.10E-08
general stress response	LBA0017	-4.8	-2.3	1000	0
kanamycin kinase	LBA1348	-4.9	-2.3	8.6	2.50E-09
hypothetical protein	LBA1802	-5.8	-2.5	13.9	1.10E-14
hypothetical protein	LBA0486	-6.6	-2.7	1000	0.00E+00
hypothetical protein	LBA1789	-6.7	-2.7	8.6	2.40E-09
hypothetical protein	LBA0490	-7.5	-2.9	1000	0.00E+00
hypothetical protein	LBA1801	-8	-3	15.5	3.20E-16
transposase	LBA1723	-10.3	-3.4	1000	0.00E+00

hypothetical protein	LBA1756	-15	-3.9	1000	0
hypothetical protein	LBA0509	-16.9	-4.1	9.8	1.60E-10
**NCK2326 vs. NCK2310 (PA vs. BoNT/A)**		**NCK2326/ NCK2310**			
hypothetical protein	LBA0888	15.3	3.9	1000	0
**NCK2310 V NCK2345 (BoNT/A V BoNT/A and PA)**		**NCK2310/ NCK2345**			
hypothetical protein	LBA0888	27.3	4.8	1000	0
aggregation promoting protein	LBA0493	4.1	2	41.8	1.50E-42
ABC transporter ATP-binding protein/permease	LBA1357	3.9	2	15.4	3.80E-16
hypothetical protein	LBA1187	-4.4	-2.1	1000	0.00E+00
ABC transporter ATP-binding protein	LBA1188	-5	-2.3	1000	0.00E+00
GntR	LBA1189	-6.1	-2.6	1000	0.00E+00
ABC transporter permease	LBA1679	-13.2	-3.7	1000	0.00E+00
ABC transporter ATP-binding protein	LBA1680	-19	-4.2	1000	0.00E+00
**NCK2345 vs. NCK2326 (BoNT/A and PA vs. PA)**		**NCK2345/ NCK2326**			
hypothetical protein	LBA1797	9.6	3.3	1000	0
ABC transporter	LBA1044	7.7	2.9	1000	0
glutamine ABC transporter ATP-binding protein	LBA1045	7.4	2.9	1000	0
ABC transporter ATP-binding protein	LBA1680	7.3	2.9	1000	0
OppA	LBA1665	7	2.8	1000	0
glutamine ABC transporter membrane spanning permease	LBA1042	5.9	2.6	34.2	6.80E-35
ABC transporter permease	LBA1679	5.5	2.5	1000	0
hypothetical protein	LBA1800	5	2.3	1000	0
glutamine ABC transporter substrate-binding protein	LBA1046	4.7	2.2	1000	0
PacL	LBA1683	-4.3	-2.1	7.5	3.20E-08

A

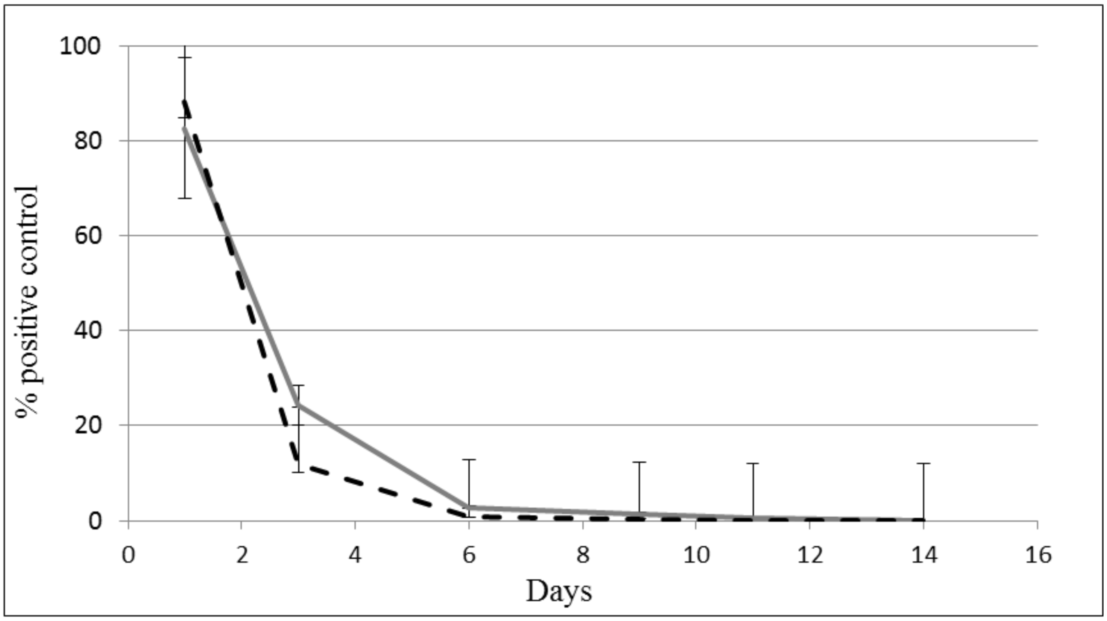

B

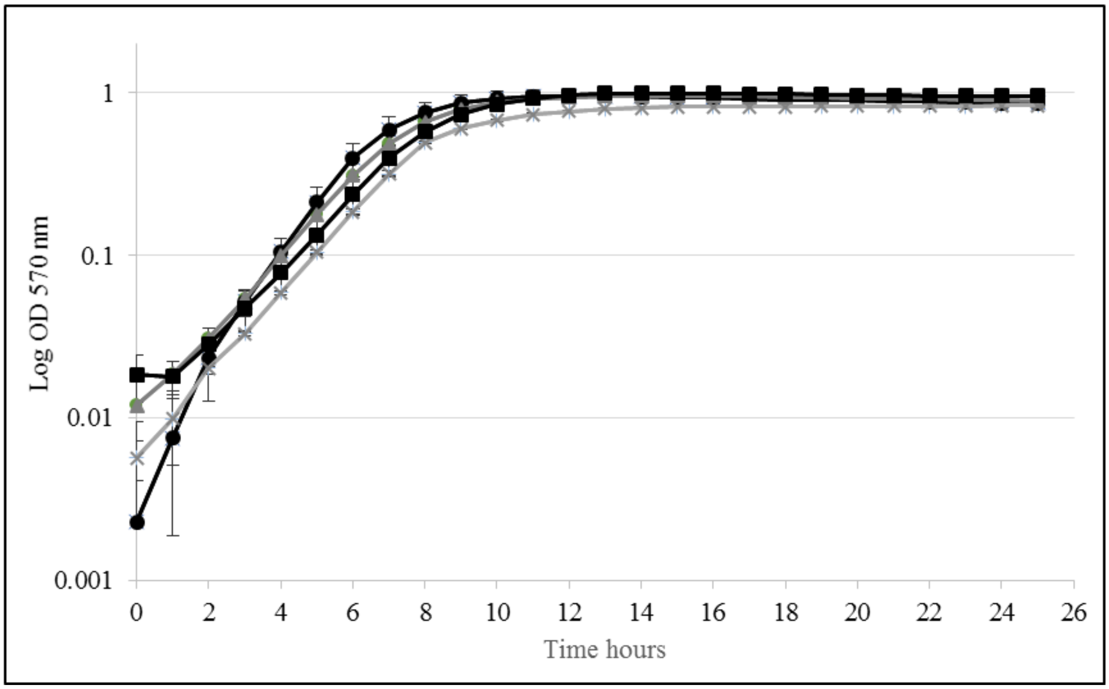

Supplemental Figure 1. Plasmid stability assays and growth curves. (A) *L. acidophilus* NCK1839 and NCK2307 were grown for 14 days and plated on MRS agar plates with and without the presence of erythromycin to determine plasmid stability of pTRK1074 expressing BoNT/A (dashed line) and pTRK896 expressing PA (black line). Error bars indicate the standard deviation of the mean. **(B)** *L. acidophilus* NCK1909 (control, black circles), NCK2310 (BoNT/A, grey triangle), NCK2326 (PA, grey X) and NCK2345 (BoNT/A and PA, black square) were grown in MRS broth for 25 hours at 37°C. Error bars indicate the standard deviation of the mean.
